# Direct-Ink-Write Printing and Electrospinning of Cellulose Derivatives for Conductive Composite Materials

**DOI:** 10.3390/ma15082840

**Published:** 2022-04-13

**Authors:** Runfeng Shi, Jiankang Zhang, Jinheng Yang, Yanglei Xu, Cuihuan Li, Sheng Chen, Feng Xu

**Affiliations:** 1 Beijing Key Laboratory of Lignocellulosic Chemistry, Beijing Forestry University, Beijing 100083, China; shirunfeng88@bjfu.edu.cn (R.S.); jiankang_zhang@bjfu.edu.cn (J.Z.); bjfuyjh@163.com (J.Y.); xuyanglei@bjfu.edu.cn (Y.X.); cuihuanli@bjfu.edu.cn (C.L.); 2 State Key Laboratory of Biobased Material and Green Papermaking, Qilu University of Technology, Shandong Academy of Sciences, Jinan 250353, China

**Keywords:** direct-ink-write print, ethyl cellulose, electrospinning, cellulose acetate, conductive composite materials

## Abstract

The aim of this study is to realize the controlled construction and modulation of micro-/nanostructures of conductive composite materials (CCMs) in a facile way. Herein, interdigital electrodes are prepared by direct-ink-write printing co-blended inks made of ethyl cellulose and carbon nanotubes on cellulose paper. The cellulose nanofibers (CFs) are prepared by electrospinning cellulose acetate on to an aluminum foil, followed by deacetylation in NaOH/ethanol. All co-blended inks exhibit a typical non-Newtonian shear thinning behavior, enabling smooth extrusion and printing. The above electrodes and the conductive CF films with excellent thermal stability are assembled into a pressure sensor, which has a high sensitivity (0.0584 KPa^−1^) to detect the change in external loading pressure. The obtained porous CFs film is further endowed with conductivity by in situ polymerization of polypyrrole (PPy), which are uniformly distributed on the CFs surface as particles; a triboelectric nanogenerator is constructed by using the CF@PPy film as a tribo-positive friction layer to achieve efficient energy harvesting (output voltage = 29.78 V, output current = 2.12 μA). Therefore, the construction of CCMs with micro-/nanostructures based on cellulose derivatives have essential application prospects in emerging high-tech fields, such as green electronics for sensing and energy harvesting.

## 1. Introduction

Conductive composite materials (CCMs) are widely used for sensing and energy devices in the field of wearable flexible electronics [1,2]. For this field, the CCMs are always composed of flexible substrates and conductive materials [3]. However, most of the existing flexible substrates used for CCMs rely on petroleum-based products such as polyurethane [4] and melamine [5], which are difficult to degrade and cause environmental pollution problems. Cellulose, as the most abundant degradable polymer in nature, has attracted tremendous attention owing to its good biocompatibility [6,7]. Cellulose also has the advantage of being widely available and low-cost, making it an ideal material for application as a flexible substrate or additive in CCMs [8], whereas the high degree of polymerization, hydrogen bonding, and van der Waals interactions of cellulose make it difficult to be dissolved in common solvents and not easy to be processed or molded [9,10]. Therefore, functionalization of cellulose by acetylation, ethylation, etherification, and so forth, to prepare the cellulose derivatives to be dissolved in common solvents, will broaden the application range of cellulose [11,12,13,14]. Cellulose derivatives are both biodegradable and easy to be processed, which makes them ideal for use as flexible substrates and/or additives for CCMs.

The micro-/nanostructure of CCMs is an essential factor affecting the working performance of various electronic devices, such as pressure sensors and triboelectric nanogenerators (TENGs) [15,16,17]. The 3D mesh-like structure with higher specific surface area is beneficial to improve the sensing performance of pressure sensors and the output performance of TENGs [18,19]. In order to achieve controlled construction and modulation of micro-/nanostructures of cellulose derivatives, direct-ink-write printing and electrostatic spinning (electrospinning) technologies are available [20,21], which are current state-of-the-art and high-efficiency additive manufacturing technologies for building 3D mesh-like structures. Extrusion-based 3D printing (direct-ink-write printing) is the most cost-effective among the 3D fabrication technologies, which can directly print out diverse sophisticated patterns [22,23]. Direct-ink-write printing allows printing of practically any material, as long as the precursor ink can be engineered to demonstrate appropriate rheological behavior. This technique acts as a unique pathway to introduce design freedom, multifunctionality, and stability simultaneously into its printed structures [24]. Direct-ink-writing printing is used in a variety of applications, such as electronics, food, and biomedical industries [25]. Blending cellulose derivatives with conductive materials can be used to prepare conductive inks for direct-ink-write printing. Carbon nanotubes (CNT) are 1D conductive materials with superior electrical and thermal conductivity [26,27]. CNT-based printing materials with favorable viscoelastic properties have demonstrated satisfactory printability [28,29]. The concentrated CNT-based inks with a sufficiently high shear elastic modulus and shear yield strength exhibit ideal gel-like behavior and superior filament shape retention after extrusion, which can build various sophisticated and accurate architectures [23,30].

Electrospinning is another technology for building 3D mesh-like structure, which has the advantage of simple operation and wide application areas [31,32]. Fibers prepared by electrospinning are characterized by their small diameter, layer thinness, high porosity, and low base weight. Hussain et al. prepared a series of fibers using the electrospinning technique, followed by electroless deposition of copper metal to obtain the conductive fibers. These conductive fibers could be used in various applications, such as wearable electronics, flexible displays, and energy storage [33,34,35]. Cellulose derivatives can be dissolved in organic solvents to prepare films with a porous network by electrospinning. Such films are difficult to be applied to CCMs due to the electrical insulation of cellulose derivatives [36]. Polypyrrole (PPy) is a conductive polymer with high electrical conductivity that is biocompatible and easily synthesized from pyrrole monomer [37,38]. PPy is widely used in electrode materials [39], sensors [40], electrochromic [41], and electromagnetic shielding [42]. However, the poor processing performance and mechanical strength of pure PPy film limit the further application of PPy. The preparation of PPy composites is an ideal improvement method [40]. Therefore, in situ polymerization of PPy on cellulose-derived film obtained by electrospinning can construct composites with both good electrical and mechanical properties.

Herein, an interdigital electrode was prepared by blending ethyl cellulose (EC) with CNT through direct-ink-write printing technology, and the rheological shear properties of EC/CNT inks during printing were investigated. The 3D mesh-like conductive composite cellulose nanofiber (CF) films were prepared by electrospinning cellulose acetate (CA) onto an aluminum foil, followed by deacetylation in NaOH/ethanol and polymerization of conductive PPy. The above interdigital electrode and porous conductive CF@PPy film were assembled into a pressure sensor and the sensing performance was evaluated. In addition, the obtained CF@PPy film was also used as a tribo-positive friction layer and the electricity generation performance of the device was tested. The construction of CCM with a 3D mesh-like structure from cellulose derivatives by direct-ink-write printing and electrospinning has important application prospects in the fields of pressure sensing and efficient energy harvesting by TENGs.

## 2. Materials and Methods

### 2.1. Materials

Ethyl cellulose (EC, 270–330 mPa·s), cellulose acetate (CA, acetyl content = 39.8 wt%), NaOH (97%), ethanol (99.7%), hydrochloric acid (HCl, 37%), sodium chloride (NaCl, 99%), and polypyrrole (PPy) were purchased from Macklin (Shanghai, China). Acetone (>99.5%) was purchased from Sigma-Aldrich (St. Louis, MO, USA). Carbon nanotube (CNT) was purchased from XF-Nano (Nanjing, China). All materials and reagents were used without further purification. Cellulose paper (basis weight = 30 g/m^2^) was purchased from Henglian Co. Ltd. (Shanghai, China).

### 2.2. Direct-Ink-Write Printing of EC/CNT Electrodes

**Figure 1:**
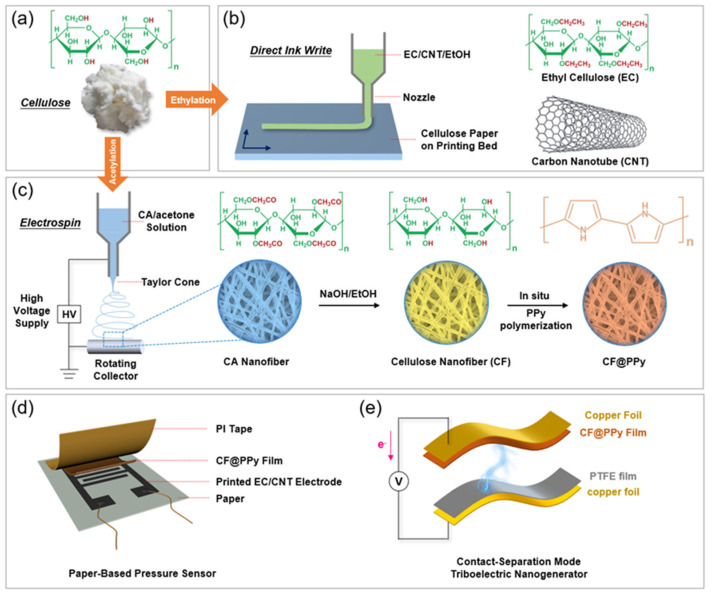
(**a**) Photo and chemical structure of cellulose. (**b**) Schematic illustration of direct-ink-write printing EC/CNT inks. (**c**) Schematic illustration of electrospinning CA, deacetylation, and in situ polymerization of PPy. (**d**) Scheme showing the structure of the paper-based pressure sensor with printed EC/CNT and CF@PPy as electrode and active layer, respectively. (**e**) Scheme showing the structure of the contact-separation TENG with CF@PPy film as tribo-positive friction layer.

As schemed by Figure 1a,b, the EC/CNT electrode was prepared by ethylating cellulose, blending EC and CNT, and direct-ink-write printing on paper. Cellulose, as a natural polymer, is difficult to be dissolved in common solvents [10], therefore the derivatization of cellulose is required. Cellulose was modified by ethylation, where the hydrogen atom in the hydroxyl group (–OH) on the cellulose unit was replaced by ethyl group (–CH_2_CH_3_) [43,44]. To prepare the conductive ink, EC and CNT were dissolved/dispersed in ethanol by vigorous stirring and the solid content was controlled to be 12 wt%. The ratio of EC and CNT in the obtained inks was 30:70, 40:60, 50:50, 60:40, and 70:30; these inks were named as EC/CNT-70, EC/CNT-60, EC/CNT-50, EC/CNT-40, and EC/CNT-30, respectively. The prepared inks were printed on a cellulose paper (basis weight = 30 g/m^2^) by a direct-ink-write printer (Yida, Shenzhen, China). The inks were sealed in a separate syringe and extruded by pressure from an air compressor; the metal nozzle (21G) and plastic nozzle (18G) were used. The patterns printed on the substrates were controlled by programmed procedures.

### 2.3. Electrospinning of CA and In Situ Polymerization of PPy

As shown in Figure 1c, CF@PPy films were fabricated by electrospinning CA, deacetylation, and in situ polymerization of PPy. CA was dissolved in a mixture of acetic acid and acetone (volume ratio = 1:1) at room temperature and the solid content was controlled to be 10 wt%. The CA solution was sealed in a syringe and spun at high voltage to obtain CA film at room temperature and relative humidity of 30%; the metal nozzle (21G) was used, and a roller receiver wrapped with aluminum foil was chosen to collect the electrospun fibers. The electrospun parameters were an applied voltage of 18 kv, a distance of 130 mm between the syringe needle and the aluminum foil, a feed speed of 0.005 mm/s, and a receiver speed of 3000 r/min, respective. The obtained CA film was deacetylated by immersion in 0.05 M NaOH/ethanol solution for 8 h, and then washed with deionized water and dried at room temperature. The dried film was immersed in pyrrole monomer solution for 5 min, and then transferred into FeCl_3_ solution (8 wt%) with 0.3 M HCl for reaction at 4 °C for 10 min. The obtained film was washed with 0.3 mol/L HCl solution, 0.1 mol/L NaCl solution and deionized water in turn, and dried at room temperature to obtain the conductive film. Finally, the conductive film was dispersed in water by the homogenizer at high speed and then filtered to obtain the CF@PPy film.

### 2.4. Preparation of Paper-Based Pressure Sensor

The direct-ink-write printed EC/CNT pattern on a flexible cellulose paper (basis weight = 30 g/m^2^) was used as the interdigital electrode. The obtained CF@PPy film was then placed onto this flexible substrate to contact the electrode and used as the piezoresistive layer. After being encapsulated by polyamide tape, the paper-based pressure sensor was obtained (Figure 1d).

### 2.5. Preparation of Triboelectric Nanogenerator

A contact-separation TENG was obtained as shown in Figure 1e. |A polymethyl methacrylate sheet was used as the substrate of TENG. CF@PPy film and polytetrafluoroethylene (PTFE) film were used as the tribo-positive and tribo-negative friction layer, respectively. Copper foils were used as the electrodes. The friction layers and electrodes were attached onto the opposite sides of the substrate face to face.

### 2.6. Characterization

The morphologies of EC/CNT electrodes, CA nanofiber film, and CF@PPy film were demonstrated by using a scanning electron microscope (SEM, Hitachi SU-8010, Tokyo, Japan). The rheological properties of EC/CNT inks were measured on the rotational rheometer (Bohlin CVO-100 901, Malvern, UK). The water contact angles of EC and EC/CNT electrodes were tested using a contact angle meter (Kino SL200KS, Boston, MA, USA). The Fourier transform infrared (FTIR) spectra of CA, deacetylated CA, and CF@PPy film were investigated by a FTIR spectrometer (Bruker Vertex 70, Karlsruhe, Germany). Thermogravimetric analysis (TGA) of EC, EC/CNT film, CA, CF@PPy film were measured by the DTG-60 from 50–800 °C. The current changes in the sensor with various pressure loadings were tested and recorded with an electrochemical working station (Autolab 302N, Herisau, Switzerland). The output performance of the TENG during applying a vertical force was measured. The output voltage and current signals were investigated using an oscilloscope (RIGOL DS1102E, Suzhou, China) and an electrochemical working station (Autolab 302N, Herisau, Switzerland), respectively.

## 3. Results and Discussion

### 3.1. Morphology, Extrusion, Rheological and Hydrophobic Properties of EC/CNT Electrodes

**Figure 2:**
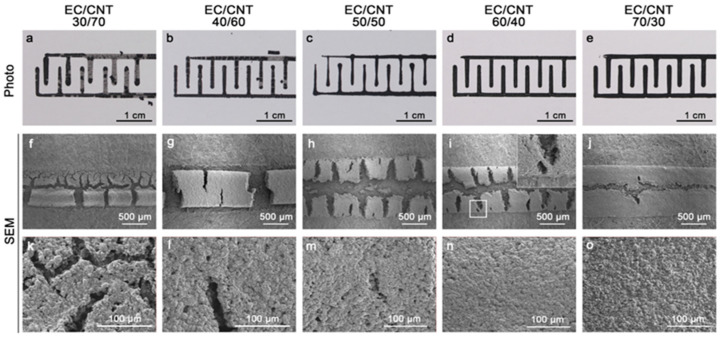
(**a**–**e**) Photo and morphology under (**f**–**j**) low and (**k**–**o**) high magnification of EC/CNT electrodes with different ratios (30/70, 40/60, 50/50, 60/40, and 70/30). The inset in (**i**) shows the connection in the electrode.

The photographs of printed electrodes with different EC and CNT ratios are shown in Figure 2a–e. The extruded inks showed obvious disconnection (Figure 2a) when the EC content was low. The coherence of the inks became better as the concentration of EC increased. The obvious cracking and complete gaps of patterns were observed for the electrodes with low EC concentration (Figure 2f–h), which would affect the electron transport. As shown in Figure 2i,j, when increasing the EC concentration, the pattern cracking was reduced and the connection was formed (inset in Figure 2i); this is crucial to the electron transportation and, thus, the conductivity of electrodes. The morphologies were observed under high magnification of SEM (Figure 2k–o), showing that the surface of the electrode became denser as the EC concentration increased. This was due to the excellent film-forming property of EC, but the CNT cannot easily form film [45]. The superior denseness would facilitate the transport of electrons and increase the conductivity of the electrode. However, too low content of CNT, which was the conductive material, could reduce the electrical conductivity of the electrodes [46,47]. Therefore, increasing the content of CNT while ensuring that the ink pattern was not disconnected were critical to the fabrication of electrodes with optimal electrical conductivity.

**Figure 3:**
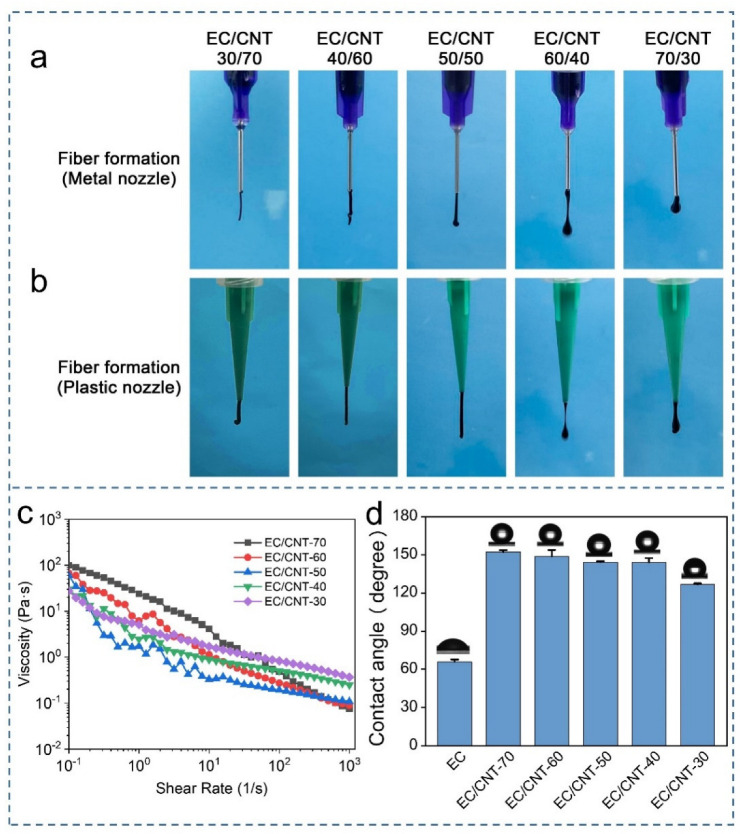
Photos of different inks/filaments during direct-ink-write printing using (**a**) metal and (**b**) plastic nozzle. (**c**) Steady-state viscosity of different inks as a function of shear rate. (**d**) The water contact angle of EC and EC/CNT electrodes.

As shown in Figure 3a, the stainless-steel needles could extrude all ratios of EC/CNT inks without clogging. When the EC content was low, the extruded inks were fibrous, which was desirable for layer stacking and might be not beneficial for direct writing. When the EC content was high, the extruded inks were the desirable teardrop shape. The liquid ink facilitated its flow over the surface of the cellulose paper (basis weight = 30 g/m^2^), which facilitated the preparation of interdigital electrodes [48]. As shown in Figure 3a, the extruded inks of EC/CNT-30 and EC/CNT-40 exhibited a teardrop shape and thus met the printing condition for paper-based electrodes. In addition, extrusion with the plastic needle tip was also possible without clogging (Figure 3b). The ink extrusion shapes were consistent with the stainless-steel needles described above, which indicated that the printing inks we prepared were suitable for the needles made of a wide range of materials. Figure 3c shows the viscosities of the inks as a function of shear rate. All the inks exhibited a typical non-Newtonian shear thinning behavior, enabling the smooth extrusion, and allowing for maintenance of the filamentary shape during direct-ink-write printing [49].

The wettability of the EC/CNT electrodes were evaluated by water contact angles. As shown in Figure 3d, the pure EC film was hydrophilic, and its contact angle was 65.9°. When a small amount of CNT was added (EC/CNT-30), the electrode became hydrophobic with a contact angle of 126.8°. As the amount of CNT added increased, the electrode exhibited superior hydrophobicity (152.3°). The hydrophobicity of the material surface is generally related to the chemical properties and the surface roughness [50]. Pure EC film had a dense and smooth surface and therefore exhibited hydrophilicity. The EC/CNT electrodes exhibited excellent hydrophobicity because CNT was hydrophobic, and the addition of CNT resulted in a cracked roughness (Figure 2k–o) on the surface.

### 3.2. Morphology, Thermal Stability, and Chemical Structure of CF@PPy Films

**Figure 4:**
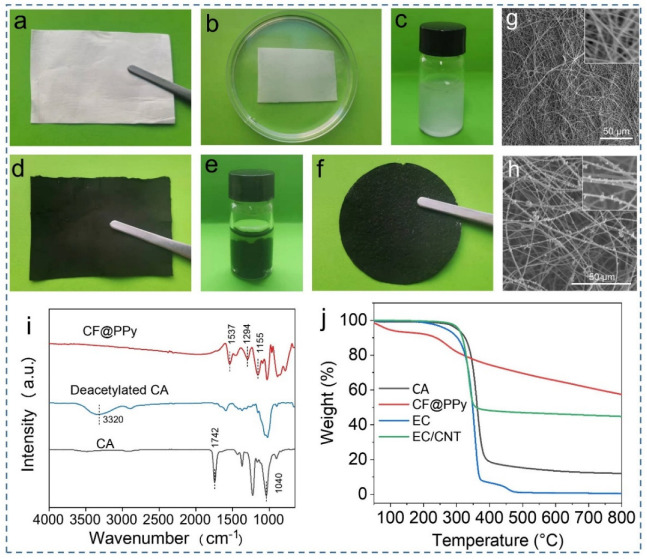
Photos of (**a**) CA film, (**b**) CA film immersion in NaOH/ethanol solution, (**c**) CA film suspension, (**d**) CA@PPy film obtained by direct polymerization of PPy on CA film, (**e**) CF@PPy suspension, and (**f**) CF@PPy film reformed by filtration. The morphology of (**g**) CA and (**h**) CF@PPy film. (**i**) FTIR spectra of CF@PPy, deacetylated CA, and CA. (**j**) TGA curves of CA, CF@PPy, EC, and EC/CNT.

Figure 4a–d show the process of in situ polymerization of PPy. The CA film had a white color (Figure 4a) as well as a porous structure and a smooth nanofiber surface (Figure 4g). After deacetylation in NaOH/ethanol (Figure 4b), the obtained film, which maintained the original porous and network structures (Figure S2a), can be redispersed into CF suspension (Figure 4c) by sonification. After polymerization of PPy, the prepared CF@PPy film had a black color (Figure 4d). As can be seen in Figure 4h, the PPy particles seem to cover the whole nanofiber surface, consequently making the surface rougher. The evenly distributed PPy particles on the nanofiber surface provided an excellent conductive path and endowed the film with good electrical conductivity. In addition, the diameter distribution of the CF@PPy is shown in Figure S3. In total, 80% of the nanofibers had a diameter ranging from 650 nm to 1450 nm. Figure 4e shows the CF@PPy suspension formed by dispersing the CF@PPy film in water by using a high-speed dissolver. The CF@PPy film can be reformed by filtering the suspension (Figure 4f). As shown in Figure S2b, the reformed CF@PPy also had a porous structure and the PPy particles were observed. The CF@PPy films with different shapes can be prepared by this approach, which is beneficial to a variety of CCMs. The mechanical properties of CA film, deacetylated CA film, CF@PPy film, and reformed CF@PPy film after filtration are shown in Figure S4. Compared with the electrospinned CA film, the deacetylated CA film and the CF@PPy film had larger tensile strength and strain, which is beneficial to their practical applications. However, after reforming the CA@PPy film by filtration, the mechanical performance was significantly reduced; this may be attributed to the shortening of nanofibers during dispersing.

To further investigate the chemical structure of CA, deacetylated CA, and CF@PPy film, their FTIR spectra were tested and are shown in Figure 4i. The characteristic peaks at 1742 and 1040 cm^−1^ were attributed to the C=O and C–O–C in CA spectrum [6]. In the deacetylated CA spectrum, the C=O at 1742 cm^−1^ was not observed and the O–H at 3320 cm^−1^ appeared, which indicated that the CA film was successfully deacetylated. After polymerizing PPy, the O–H at 3320 cm^−1^ and the C–H at 2990 cm^−1^ were significantly weakened in CF@PPy film spectrum, which indicated that the cellulose fiber was covered by PPy [51,52]. The peaks at 1537, 1294, and 1155 cm^−1^ were attributed to C–C of pyrrole ring, C–H, and C–N in-plane deformation modes, which were the characteristic peaks of PPy [51]. The XRD patterns of original CA powder, electrospun CA, deacetylated CA, and CF@PPy are presented in Figure S5. For the original CA power, the intense diffraction peaks centered at 2θ = 8.6°, 10.5°, 13.3°, 17.3°, and 21.3° revealed the crystalline nature of CA power, while for the electrospun CA, both crystalline and amorphous phases were observed, indicating its semi-crystalline nature [53]. This is due to that the dissolving in acetone caused the disorderliness of CA power before electrospinning [54]. The deacetylating caused no obvious changes in the crystalline structures when compared the XRD patterns of electrospun CA and deacetylated CA. After polymerization of PPy, the obtained CF@PPy exhibited the broad peaks related to the scattering from PPy chains at the interplanar spacing [55].

We also measured the thermal stability of the films before and after polymerization of PPy. As shown in Figure 4j, the main decomposition temperature of CA film was 290 °C to 400 °C and the mass loss of this process reached more than 70%, due to the decomposition of CA into small molecules. The starting decomposition temperature of CF@PPy film was 205 °C that was lower than that of CA film, which might be due to the destruction of the crystalline structure and hydrogen bonding of CA after polymerization of PPy. The major decomposition compounds were small molecules CA and pyrrole. The maximum decomposition rate temperatures of both were similar (Figure S1). Although the thermal stability of CF@PPy film decreased, the starting decomposition temperature was still above 200 °C, which could meet the requirement of thermal stability for most applications. After the temperature reached 800 °C, the CF@PPy film still had a larger residual mass compared to the CA film. This residue was mainly the incomplete decomposition of PPy. The main decomposition temperatures were almost identical for EC and EC/CNT. However, the residual mass of EC/CNT was larger than that of EC due to the incomplete decomposition of the CNT.

### 3.3. Sensing and Electrical Generation Performance of CCMs

**Figure 5:**
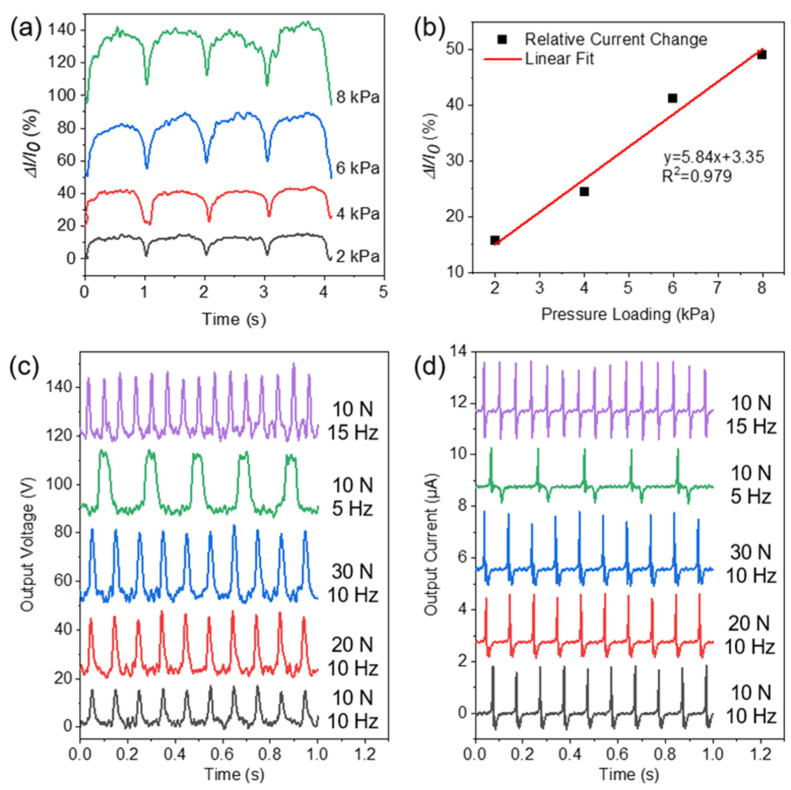
(**a**) The relative current changes in the sensor under different pressures. (**b**) The peak relative current change in the sensor under pressure loadings of 2–8 KPa. (**c**) Output voltage and (**d**) output short-circuit current of the TENG with various external press forces and frequencies.

The cellulose derivative-based CCMs we prepared were further assembled into a pressure sensor and a contact-separation TENG. The relative current changes (ΔI/I_0_, where ΔI is the current change and I_0_ is the current of the sensor in the unloading state) in the pressure sensor under different pressures in cyclic loading-unloading were further measured (Figure 5a). The maximum current change in this sensor increased monotonically with the increased pressure loading from 2 to 8 KPa. In addition, the intensity of sensing signals was relatively stable over multiple cycles, indicating that our prepared sensor had good reliability. To further evaluate the working performance of the pressure sensor, sensitivity (S) was introduced, which indicated the sensor response to changes in pressure loadings [56]. To calculate the sensitivity, the maximum current change values under the pressure range of 2–8 KPa at a fixed voltage (3 V) were measured. The relative current increase rate of the sensor remained consistent as the pressure increased from 2 to 8 KPa (Figure 5b). A linear fit to the obtained curve was performed to calculate the slope (i.e., sensitivity). The sensitivity of the sensor was calculated as 0.0584 KPa^−1^ according to S = Δ(ΔI/I0)/ΔP, where ΔP is the change in the applied pressure. This relatively high sensitivity (Table S1) would endow the sensor with promising potentials for diverse applications.

Figure 5c exhibits the output voltage of the TENG with various external press loading and frequencies. The peak output voltage of the TENG increased significantly as the external force increased from 10 N to 30 N [57]. Since the TENG could generate different electrical signals for different loading, the TENG could be also applied as a self-powered pressure sensor [58]. The frequency of output voltage signals of the TENG increased as the press frequency increased from 5 Hz to 15 Hz, but there was no obvious change in the peak output voltage. Figure 5d shows the output short-circuit current of the TENG under various external press loading and frequencies. As the external force increased from 10 N to 30 N, there was no significant change in the peak output short-circuit current. As the press frequency increased from 5 Hz to 15 Hz, the frequency of the output short-circuits current of the TENG increased but there was no obvious change in the peak output short-circuit current. Therefore, the external force and frequencies are critical to the output voltage and current of the TENGs, respectively [59].

## 4. Conclusions

In this work, the rheological shear properties of the EC and CNT co-blended inks and the morphology of the electrodes were investigated. Additionally, the morphology and thermal stability of CF@PPy film were evaluated.

(1) The surface of the electrodes became denser and formed a conductive path as the EC concentration increased. All the inks exhibited a typical non-Newtonian shear thinning behavior, enabling smooth extrusion and printing.
(2) The PPy particles were uniformly distributed on the nanofiber surface, providing an excellent conductive path. The film started to decompose above 200 °C, which could meet the requirement of thermal stability for most applications.
(3) The above electrodes and CF@PPy film were assembled into a pressure sensor, which could indicate the change in pressure and had a high sensitivity (0.0584 KPa^−1^).
(4) TENG was constructed by using CF@PPy film as a tribo-positive friction layer, which could achieve a high electrical energy output (voltage = 29.78 V, current = 2.12 μA).

## Data Availability

All the data is available within the manuscript.

## Supplementary Materials

The following supporting information can be downloaded at: https://www.mdpi.com/article/10.3390/ma15082840/s1. Figure S1: DTG curves of CA, CF@PPy, EC, and EC/CNT., Figure S2: SEM images of the (a) deacetylated CA film and (b) reformed CF@PPy film after filtration, Figure S3: Diameter distribution of CF@PPy, Figure S4: Mechanical property of the CA film, deacetylated CA film, CF@PPy film, and reformed CF@PPy film after filtration, Figure S5: XRD patterns of the original CA powder, electrospun CA, deacetylated CA, and CF@PPy, Table S1: Comparison of the pressure sensor in our work with others in the literature. References [60,61,62,63,64,65,66,67,68,69] are cited in the Supplementary Materials.
