# Supplementary material for: Direct-Ink-Write Printing and Electrospinning of Cellulose Derivatives for Conductive Composite Materials
Source: Materials (Basel). 2022 Apr 13;15(8):2840. doi: 10.3390/ma15082840 (PMC9028373; doi:10.3390/ma15082840)
Supplement: Supplementary file 1 [file materials-15-02840-s001.zip › materials-1656179-supplementary.pdf]

Supporting Information

Direct-Ink-Write Printing and Electrospinning of Cellulose Derivatives for Conductive Composite Materials

Runfeng Shi[1,†], Jiankang Zhang[1,†], Jinheng Yang[1], Yanglei Xu[1], Cuihuan Li[1], Sheng Chen[1,*], Feng Xu[2,*]

[1] Beijing Key Laboratory of Lignocellulosic Chemistry, Beijing Forestry University, Beijing 100083, China; 1196181303@qq.com (R.S.); jiankang_zhang@bjfu.edu.cn (J.Z.); bjfuyjh@163.com (J.Y.); xuyanglei@bjfu.edu.cn (Y.X.); cuihuanli@bjfu.edu.cn (C.L.)

[2] State Key Laboratory of Biobased Material and Green Papermaking, Qilu University of Technology, Shandong Academy of Sciences, Jinan 250353, China

[*] Correspondence: shengchen@bjfu.edu.cn (S.C.); xfx315@bjfu.edu.cn (F.X.)

[†] These authors contributed equally to this work.

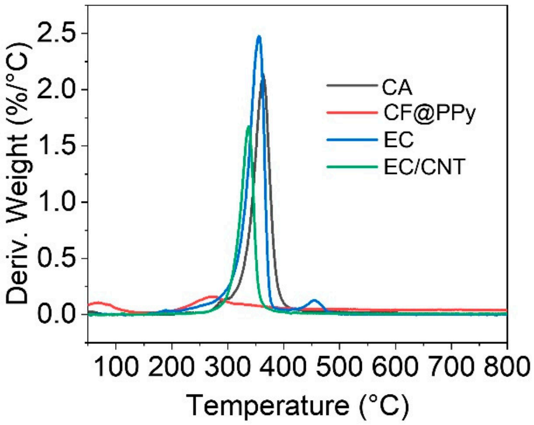

Figure S1. DTG curves of CA, CF@PPy, EC, and EC/CNT.

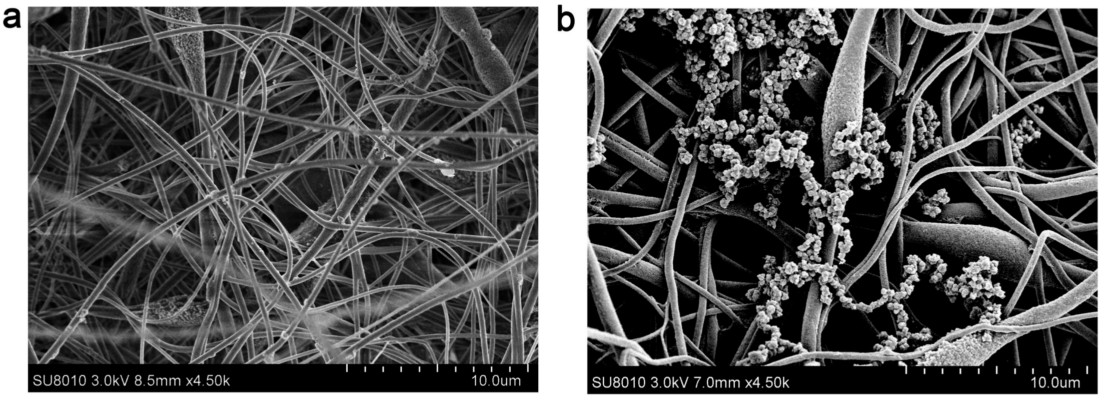

Figure S2. SEM images of the (a) deacetylated CA film and (b) reformed CF@PPy film after filtration.

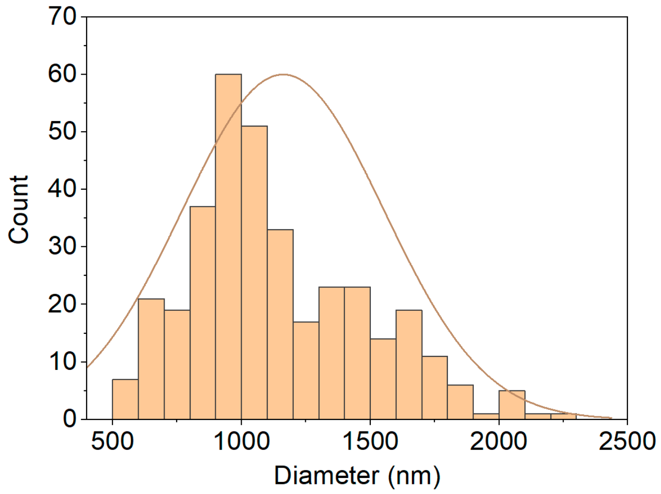

Figure S3. Diameter distribution of CF@PPy.

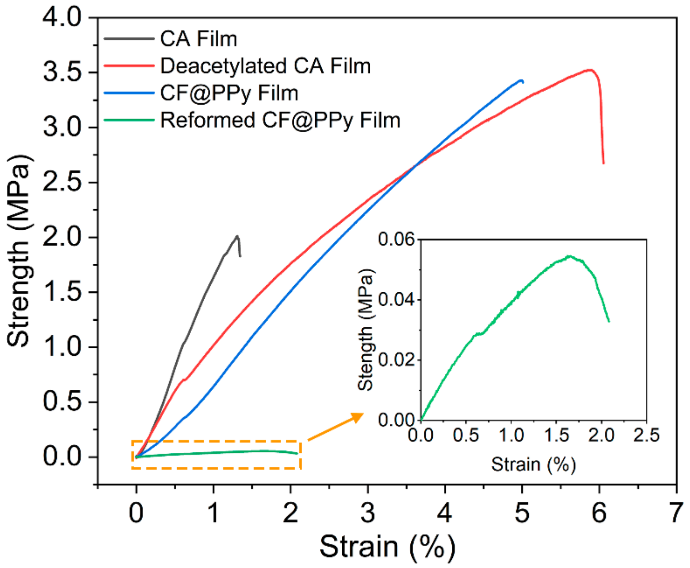

Figure S4. Mechanical property of the CA film, deacetylated CA film, CF@PPy film, and reformed CF@PPy film after filtration.

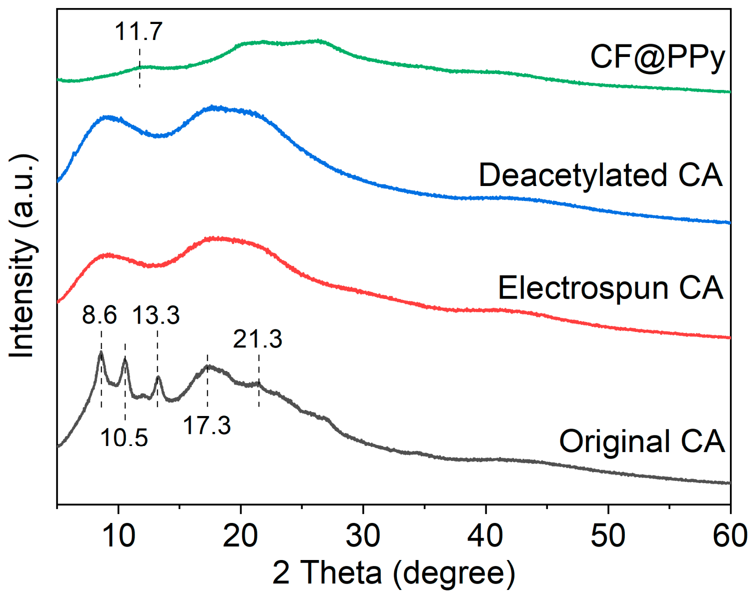

Figure S5. XRD patterns of the original CA powder, electrospun CA, deacetylated CA, and CF@PPy.

Table S1. Comparison of the pressure sensor in our work with others in literature.

References	Materials	Sensitivity (KPa^{-1})	Pressure range (KPa)
Our work	Ethyl cellulose and polypyrrole	0.0584	2-8
[60]	Polyurethane sponge and reduced graphene oxide	0.03	2-10
[61]	Polyaniline sponge, reduced graphene oxide	0.042-0.152	0-27
[62]	Graphene/polyimide foam	0.023	3.5-6.5
[63]	Graphene/carbon nanotube foam	0.02	2.5-13
[64]	Polyvinyl alcohol and polyacrylamide	0.02	3.27-6.83
[65]	Polyacrylamide and polyaniline	0.05	1-10
[66]	SiO_2 nanofibers and alginate	0.24	2.5-6
[67]	Graphene sheet and Cu/Au electrodes	0.0045	0-50
[68]	Graphene foam	0.027	0-50
[69]	CNT/graphene	0.27	0.3-6

References

1. Yao, H.B.; Ge, J.; Wang, C.F.; Wang, X.; Hu, W.; Zheng, Z.J.; Ni, Y.; Yu, S.H. A Flexible and Highly Pressure-Sensitive Graphene-Polyurethane Sponge Based on Fractured Microstructure Design. *Adv. Mater.* **2013**, *25*, 6692–6698.
2. Ge, G.; Cai, Y.; Dong, Q.; Zhang, Y.; Shao, J.; Huang, W.; Dong, X. A Flexible Pressure Sensor Based on RGO/Polyaniline Wrapped Sponge with Tunable Sensitivity for Human Motion Detection. *Nanoscale* **2018**, *10*, 10033–10040.
3. Qin, Y.; Peng, Q.; Ding, Y.; Lin, Z.; Wang, C.; Li, Y.; Xu, F.; Li, J.; Yuan, Y.; He, X.; Li, Y. Lightweight, Superelastic, and Mechanically Flexible Graphene/Polyimide Nanocomposite Foam for Strain Sensor Application. *ACS Nano* **2015**, *9*, 8933–8941.
4. Kuang, J.; Dai, Z.; Liu, L.; Yang, Z.; Jin, M.; Zhang, Z. Synergistic Effects from Graphene and Carbon Nanotubes Endow Ordered Hierarchical Structure Foams with a Combination of Compressibility, Super-Elasticity and Stability and Potential Application as Pressure Sensors. *Nanoscale* **2015**, *7*, 9252–9260.
5. Ge, G.; Zhang, Y.; Shao, J.; Wang, W.; Si, W.; Huang, W.; Dong, X. Stretchable, Transparent, and Self-Patterned Hydrogel-Based Pressure Sensor for Human Motions Detection. *Adv. Funct. Mater.* **2018**, *28*, 1802576.
6. Duan, J.; Liang, X.; Guo, J.; Zhu, K.; Zhang, L. Ultra-Stretchable and Force-Sensitive Hydrogels Reinforced with Chitosan Microspheres Embedded in Polymer Networks. *Adv. Mater.* **2016**, *28*, 8037–8044.
7. Si, Y.; Wang, L.; Wang, X.; Tang, N.; Yu, J.; Ding, B. Ultrahigh-Water-Content, Superelastic, and Shape-Memory Nanofiber-Assembled Hydrogels Exhibiting Pressure Responsive Conductivity. *Adv. Mater.* **2017**, *29*, 1–7.
8. Mohammad Haniff, M.A.S.; Muhammad Hafiz, S.; Wahid, K.A.A.; Endut, Z.; Wah Lee, H.; Bien, D.C.S.; Abdul Azid, I.; Abdullah, M.Z.; Ming Huang, N.; Abdul Rahman, S. Piezoresistive Effects in Controllable Defective HFTCVD Graphene-Based Flexible Pressure Sensor. *Sci. Rep.* **2015**, *5*, 1–10.
9. Yang, J.; Li, X.; Lu, X.; Bao, W.; Chen, R. Three-Dimensional Interfacial Stress Sensor Based on Graphene Foam. *IEEE Sens. J.* **2018**, *8*, 1.
10. Jian, M.; Xia, K.; Wang, Q.; Yin, Z.; Wang, H.; Wang, C.; Xie, H.; Zhang, M.; Zhang, Y. Flexible and highly sensitive pressure sensors based on bionic hierarchical structures. *Adv. Funct. Mater.* **2017**, *27*, 1606066.
